# Dynamics of Hepatitis B Virus Pregenomic RNA in Chronic Hepatitis B Patients With Antiviral Therapy Over 9 Years

**DOI:** 10.3389/fmed.2022.851717

**Published:** 2022-04-29

**Authors:** Jiali Pan, Yu Tian, Jinghang Xu, Hao Luo, Ning Tan, Yifan Han, Qian Kang, Hongyu Chen, Yuqing Yang, Xiaoyuan Xu

**Affiliations:** ^1^Department of Infectious Diseases, Peking University First Hospital, Beijing, China; ^2^Department of Gastroenterology, Peking University First Hospital, Beijing, China; ^3^Department of Gastroenterology, National Center of Gerontology, Beijing Hospital, Institute of Geriatric Medicine, Chinese Academy of Medical Sciences, Beijing, China

**Keywords:** chronic hepatitis B, PgRNA, hepatitis B virus, dynamics, transcription

## Abstract

Serum hepatitis B virus pregenomic RNA (HBV pgRNA) is a potential biomarker that is correlated with covalently closed circular DNA. The long-term dynamics of HBV pgRNA in patients with chronic hepatitis B need to be explored. One hundred naïve nucleos(t)ide analog-treated patients with chronic hepatitis B were enrolled to analyze the dynamics of HBV pgRNA over 9 years. The positive rates of HBV pgRNAs declined gradually and showed biphasic kinetics. Serum HBV pgRNA levels in patients treated with entecavir became negative later than those treated with adefovir or lamivudine. Patients who remain positive for HBV pgRNA after 9 years of treatment may have higher viral transcription efficiencies. The reverse transcription efficiency of hepatitis B e-antigen (HBeAg)-positive patients was higher than that of HBeAg-negative patients at baseline and showed no difference after 24-week nucleos(t)ide analog treatment. The trajectory of serum HBV pgRNA-negative transformation differs in patients with different characteristics. Long-term dynamic monitoring of serum HBV pgRNA levels has significance in hepatitis B treatment.

## Introduction

Despite advances in diagnosis and treatment, hepatitis B virus (HBV) infection is a global health issue and is difficult to completely eliminate because of its covalently closed circular DNA (cccDNA) (1). For chronic hepatitis B (CHB) infection, current therapies include nucleos(t)ide analogs (NAs) and interferons (2). NAs require long-term, uninterrupted use. The combined use of interferons and NAs results in 3–7% hepatitis B surface antigen (HBsAg) clearance; however, there are some side effects (3). In patients receiving long-term antiviral therapy, a new marker is necessary to monitor the effects of antiviral treatment.

Serum HBV pregenomic RNA (pgRNA) is the main component of serum HBV RNA and is derived from hepatic cccDNA. Serum HBV pgRNA is encapsidated, and its detection is relatively stable (4, 5). Serum HBV pgRNA can indicate viral transcription activity to some extent (6) and can be used as a novel marker of HBV infection for antiviral treatment efficacy,

long-term prognosis, and viral rebound after treatment cessation (5–7). Liu revealed the dynamics of HBV pgRNA throughout 5 years in patients who were treated with telbivudine with or without adefovir; results showed that the dynamics were biphasic (8). In our previous study, patients treated with entecavir (ETV), adefovir (ADV), or lamivudine (LVD) were analyzed. In summary, 28.95–45.10% of CHB patients still tested positive for HBV pgRNA after 5-year antiviral treatment (8, 9). In this study, we expanded the population and prolonged the follow-up time to 9 years to describe the dynamics of HBV pgRNA in CHB patients.

## Patients and Methods

### Patients

A total of 110 CHB patients who were naïve to NA treatment in March 2007 at Peking University First Hospital (China) were evaluated. Written informed consent was obtained from all the patients. We excluded patients with decompensated liver cirrhosis, liver cancer, or transplantation. Patients with HIV or HCV co-infection and those without serum retention were excluded. Finally, 100 patients were enrolled to analyze the dynamics of HBV pgRNA over 9 years.

### Serological Marker Assays

Serological markers were assessed every 12 weeks. Alanine aminotransferase (ALT) levels were assessed using an automatic biochemical analyzer. The COBAS TaqMan assay (Roche Diagnostics, Basel, Switzerland) was used to determine the load of HBV DNA. Quantitative HBsAg and hepatitis B e-antigen (HBeAg) status were assessed using ELISA kits (Abbott Laboratories, Chicago, IL, United States). ALT levels below 50 IU/L in men and below 40 IU/L in women were considered normal. HBV DNA loads of less than 1,000 were considered below the lower limit of detection.

### Serum Hepatitis B Virus Pregenomic RNA Quantification

As described in previous studies (7, 9), serum HBV pgRNA was extracted using a QIAamp Viral Mini Kit (Qiagen, Germany). Interfering DNA was degraded using an RNase-Free DNase Set (Qiagen, Germany) for extraction. Specific HBV pgRNA primers were synthesized by the Beijing Genomics Institute; these sequences are shown in Supplementary Table 1. A RevertAid First Strand cDNA Synthesis Kit (Thermo Fisher Scientific, MA, United States) was used for reverse transcription. To quantify HBV pgRNA, quantitative real−time PCR (RT-qPCR) was performed using a TaqMan probe and an ABI Prism 7500 Sequence Detection System (Applied Biosystems, CA, United States). The mixture for RT-qPCR was denatured at 95°C for 10 min, then underwent 40 cycles at 95°C for 15 s and 60°C for 1 min. The limit of detection (LOD) was 2.30 log10 copies/mL.

### Statistical Analyses

Categorical variables and continuous variables are presented as percentages and mean ± standard deviation, respectively. Independent sample tests and Mann–Whitney *U*-tests were used for continuous variables, whereas Pearson’s chi-square test and Fisher’s exact test were used for categorical variables. A *p*-value < 0.05 was considered statistically significant. Statistical analyses were performed using IBM SPSS Statistics version 25 (SPSS, Chicago, IL, United States). Graphs were analyzed using GraphPad Prism (version 8.0, San Diego, CA, United States). Adobe Illustrator (version 24.0, Mountain View, CA, United States) was used to edit the figures.

## Results

### Clinical Characteristics and Dynamic Changes in Hepatitis B Virus Pregenomic RNA of Patients

**FIGURE 1:**
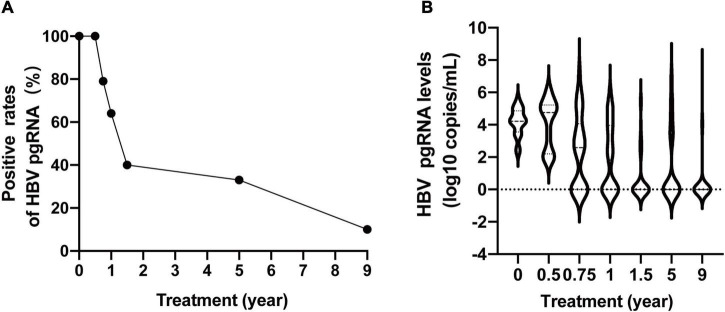
Dynamic change of serum HBV pgRNA positive rates **(A)** and levels **(B)** during 9 years of antiviral therapy.

The baseline clinical characteristics of the 100 CHB patients are shown in Table 1. The baseline average levels of ALT, HBV DNA, HBsAg, and HBV pgRNA were 161 IU/L, 7.70 log10 IU/mL, 3.72 log10 IU/mL, and 2.49 log10 copies/mL, respectively. Eighty percent of the patients tested positive for HBeAg. The positive rates of HBV pgRNA at baseline, 24, 36, 48, and 72 weeks, and 5 and 9 years were 100% (66/66), 100% (66/66), 79% (61/77), 64% (50/78), 40% (25/63), 33% (24/73), and 10% (7/71), respectively (Figure 1A). There was no significant change from baseline to 24 weeks. Subsequently, we observed a rapid decline from 24 to 72 weeks, followed by a slow decline to year 9. HBV pgRNA levels showed a biphasic decrease, as shown in Figure 1B.

**TABLE 1:** Baseline characteristics of total patients.

	Total (*n* = 100)
Male (*n*)	80 (80.0%)
Age (year)	51 ± 11.21
**Treatment**	
ETV	55
ADV	36
LVD	9
ALT(IU/L)	161 ± 162.53
HBeAg positive (n)	80 (80.0%)
HBV DNA (log10 IU/mL)	7.70 ± 1.45
HBsAg (log10 IU/mL)	3.72 ± 0.7
HBV pgRNA (log10 copies/mL)	2.49 ± 2.15

### Dynamics Changes of Hepatitis B Virus Pregenomic RNA in Different Groups

**FIGURE 2:**
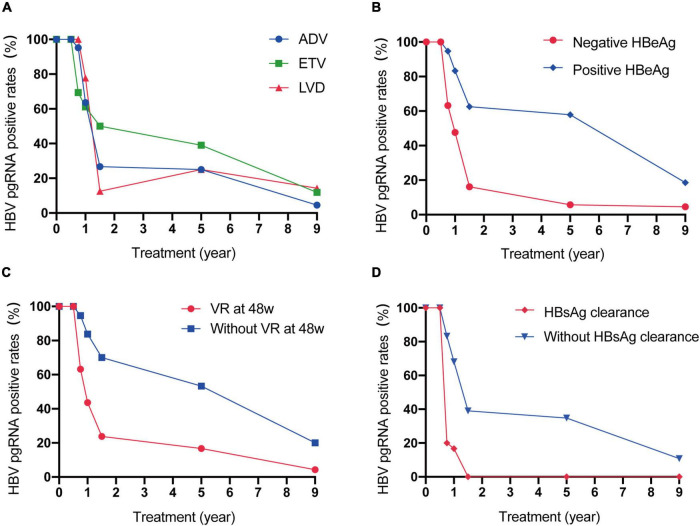
Dynamic change of serum HBV pgRNA positive rates between patients with different therapy **(A)**, with or without positive HBeAg at baseline **(B)**, with or without VR at 48w **(C)** and with or without HBsAg clearance **(D)** during 9 years of antiviral therapy.

We divided the 100 patients into different groups and observed the dynamic changes (Figure 2). The HBV pgRNA-positive rates in patients treated with ETV declined more slowly than those in patients treated with ADV or LAM. The HBV pgRNA-positive rates declined much faster in patients with negative HBeAg, virological response (VR) at 48 weeks, and HBsAg clearance. All serum HBV pgRNAs became negative within 72 weeks of NA therapy in patients with HBsAg loss. Changes in HBV pgRNA levels are detailed in Supplementary Table 2.

### Differences Between Hepatitis B Virus Pregenomic RNA-Positive and -Negative Patients at Year 9

**FIGURE 3:**
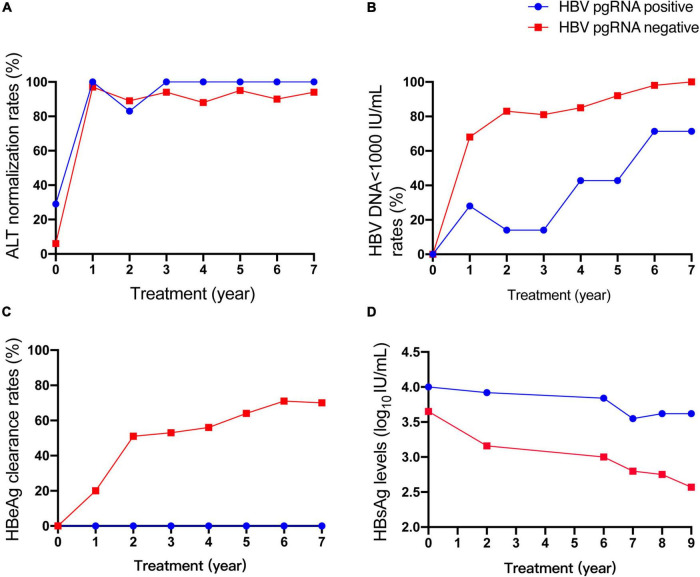
Dynamic change of ALT normalization rates **(A)**, HBV<1000 IU/mL rates **(B)**, HBeAg clearance rates **(C)**, HBsAg levels **(D)** between HBV pgRNA positive and negative patients during 9 years of antiviral therapy.

Based on the available serum, 71 CHB patients were enrolled to analyze the HBV pgRNA status with 9-year antiviral treatment. HBV pgRNA remained positive in seven (9.9%) patients after 9 years of NA treatment. Among these pgRNA-positive patients, three acquired partial virological responses, of whom two used ETV and one used LVD. One patient who was treated with ETV occurred a virological breakthrough and afterward added ADV in the third year and discontinued use in the seventh year. Other patients did not switch to other medications during the 9 years of antiviral treatment. Baseline characteristics showed no differences in sex, age, therapy, HBV DNA load, or levels of ALT, HBsAg, and HBeAg (Table 2). However, patients who were HBV pgRNA-positive showed higher HBV DNA loads and HBsAg levels at baseline. The characteristics at week 48 showed no significant differences (Supplementary Table 3). Dynamic changes in the ALT normalization rate, HBV < 1,000 IU/mL rate, HBeAg clearance rate, and HBsAg levels between HBV pgRNA-positive and -negative patients over 9 years of antiviral therapy are shown in Figure 3. No significant differences were observed in ALT normalization. However, the HBV DNA LOD rates in HBV pgRNA-positive patients were much higher and HBsAg levels were much lower than those in patients negative for HBV pgRNA. No HBV pgRNA-positive patients achieved HBeAg clearance, and HBeAg clearance rates increased gradually in patients negative for HBV pgRNA.

**TABLE 2:** Baseline characteristics of HBV pgRNA positive and negative patients after 9-year treatment.

	Total (*n* = 71)	HBV pgRNA negative (*n* = 64)	HBV pgRNA positive (*n* = 7)	*P*-value
Male (n)	57 (80.3%)	49 (76.6%)	7 (100%)	>0.05
Age (year)	51 ± 11.39	51 ± 10.62	50 ± 7.33	>0.05
Treatment				>0.05
ETV	42	37	5	
ADV	22	21	1	
LVD	7	6	1	
ALT (IU/L)	165 ± 126.61	165 ± 125.92	162 ± 142.99	>0.05
HBeAg positive (n)	51 (76.1%)	45 (70.3%)	6 (85.7%)	>0.05
HBV DNA (log10 IU/mL)	7.34 ± 1.55	7.27 ± 1.53	8.00 ± 1.69	>0.05
HBsAg (log10 IU/mL)	3.67 ± 0.72	3.65 ± 0.74	4.00 ± 0.34	>0.05

*ETV, entecavir; ADV, adefovir; LAM, lamivudine; ALT, alanine aminotransferase; HBV pgRNA, hepatitis B virus pregenomic RNA.*

### Dynamics of Transcription Efficiency Using Serum Hepatitis B Virus Pregenomic RNA

**FIGURE 4:**
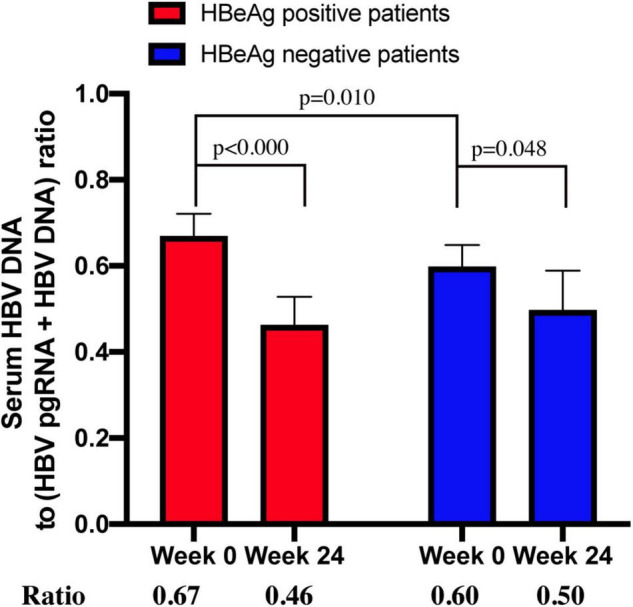
The ratio of serum HBV DNA to (HBV DNA+HBV RNA) in HBeAg-positive and HBeAg-negative CHB patients at week 0 and week 24.

Transcription efficiency was evaluated using the ratio of HBV DNA to HBV DNA + HBV pgRNA (10). The median ratios were 0.67 (*n* = 25) and 0.46 (*n* = 21) for CHB patients positive for HBeAg at week 0 and 24, respectively. The median ratios of HBeAg-negative patients at week 0 and 24 were 0.60 (*n* = 5) and 0.50 (*n* = 4), respectively (Figure 4).

The ratio between patients who were positive and negative for HBeAg at week 0 was significantly different (*p* = 0.010). The ratio between week 0 and 24 in HBeAg-positive and -negative patients (*p* < 0.000 and *p* < 0.048, respectively) was significantly different, whereas the ratio between HBeAg-positive and -negative patients at week 24 was not significantly different (*p* = 0.335).

## Discussion

The dynamics of serum HBV pgRNA during the 9-year antiviral treatment in NA-naïve CHB patients are described in this study. Similar to previous studies (8), the positive rates and levels of HBV pgRNA showed biphasic kinetics over 9 years, with rapid decline before 72 weeks and slower decline until year 9. We also analyzed the declining trend in HBV pgRNA-positive rates with respect to different clinical characteristics. Unexpectedly, patients treated with ETV appeared to have a late turning rate. Reverse transcription inhibition was likely not as effective as that of ADV and LAM. Patients with negative HBeAg, and who achieved virological response may have lower viral transcription efficiency and, therefore, more rapid decrease rates. For patients who achieved HBsAg loss, serum HBV pgRNA became negative very early, which may indicate a lower viral transcription efficiency. Regarding the turning time of HBsAg and HBV pgRNA, all HBV pgRNA disappeared before or at HBsAg loss, with a lower LOD of HBV pgRNA. However, Wu et al. reported that 65% of patients with HBV pgRNA had a later negative turning than HBsAg (11).

After 9-year NA treatment, 9.9% of the patients still had detectable serum HBV pgRNA levels. Wu et al. (11) reported that baseline serum HBV pgRNA alone was not sufficient to predict the trajectory of HBV pgRNA. Similarly, clinical characteristics were not significantly different between patients positive and negative for HBV pgRNA at the early stage of therapy. However, the difference between the two groups gradually became clear during long-term NA treatment. HBV pgRNA-positive patients had lower HBV DNA LOD rates and HBsAg levels, but no HBeAg clearance, which corresponded to higher viral transcription efficiency.

Mak et al. (12) assessed transcription efficiency using the ratio of HBV RNA/HBV DNA and found that the difference between patients positive and negative for HBeAg was noteworthy. Liao et al. (10) used the ratio of HBV DNA to HBV RNA + HBV DNA to evaluate transcription efficiency and found that HBeAg-positive patients showed lower viral transcription efficiencies than HBeAg-negative patients. As the main HBV RNA in the serum was HBV pgRNA, we assessed transcription efficiency through the ratio of HBV DNA to HBV pgRNA + HBV DNA. Nevertheless, the results of our study were different from those of Liao et al. In our study, untreated HBeAg-positive patients had a higher reverse transcription efficiency than that of HBeAg-negative patients. HBeAg-positive patients had a higher viral load at baseline than that of HBeAg-negative patients. Therefore, the ratio of HBeAg-positive patients is mathematically higher. HBeAg-negative patients had less intrahepatic cccDNA than HBeAg-positive patients, and the virus had impaired productivity (13, 14). When a virus evolves to achieve immune escape, it must abandon some infectivity (15). We also analyzed the reverse transcription efficiency after 24 weeks of treatment. Transcription efficiency was distinctly inhibited in all CHB patients, especially in those who tested positive for HBeAg. However, after 24 weeks of therapy, the difference between HBeAg-positive and -negative patients was no longer significant.

Our study has some limitations. First, the sample size was small owing to the lack of serum. Second, genotypes were not analyzed, but Chinese people are generally of the B or C type. Moreover, more accurate methods for HBV pgRNA detection are required.

In conclusion, the trajectory of serum HBV pgRNA differs in patients with different characteristics. Dynamic monitoring of serum HBV pgRNA in the long term is important.

## Data Availability Statement

The raw data supporting the conclusions of this article will be made available by the authors, without undue reservation.

## Ethics Statement

The studies involving human participants were reviewed and approved by the Ethics Committee of Shanghai Jing’an Central Hospital, Institutional Review Board of Peking University First Hospital, Peking University First Hospital. The patients/participants provided their written informed consent to participate in this study.

## Author Contributions

JP designed, performed the study, acquired, analyzed the data, and wrote the article. YT designed the study, analyzed the data, and revised the article. JX designed the research and acquired the data. HL, NT, YH, QK, HC, and YY acquired the data. XX edited, reviewed, and approved the final article. All authors contributed to the article and approved the submitted version.

## Conflict of Interest

The authors declare that the research was conducted in the absence of any commercial or financial relationships that could be construed as a potential conflict of interest.

## Publisher’s Note

All claims expressed in this article are solely those of the authors and do not necessarily represent those of their affiliated organizations, or those of the publisher, the editors and the reviewers. Any product that may be evaluated in this article, or claim that may be made by its manufacturer, is not guaranteed or endorsed by the publisher.
