# Supplementary material for: Dynamics of Hepatitis B Virus Pregenomic RNA in Chronic Hepatitis B Patients With Antiviral Therapy Over 9 Years
Source: Front Med (Lausanne). 2022 Apr 29;9:851717. doi: 10.3389/fmed.2022.851717 (PMC9099358; doi:10.3389/fmed.2022.851717)
Supplement: Supplementary file 1 [file Data_Sheet_1.pdf]

Supplementary Table 1 Specific primers of quantification for HBV pgRNA

Specific RT Primer	5′-ATTCTCAGACCGTAGCACACGACACCGAGATTGAGATCTTCTGCGCGAC-3′
Forward primer for RT-qPCR	5′-AYAGACCATCAAATGCCC-3′
Reverse primer for RT-qPCR	5′-ATTCTCAGACCGTAGCACACGACACCGAGATTGAGATCTTCTGCGCGAC-3′
TaqMan Probe	5′-FAM-CTTATCAACACTTCCGGARACTACTGTTGTTAGAC-BHQ1-3′

Supplementary Table 2 The changes of HBV pgRNA levels in different groups

**HBV pgRNA levels (log10 copies/mL)**	**Baseline**	**24w**	**36w**	**48w**	**72w**	**5y**	**9y**
**Treatment**							
ETV	4.20±0.86	4.15±1.46	2.32±2.12	0.95±1.90	1.01±1.72	1.76±2.40	0.52±1.49
ADV	4.21	2.05	3.65±3.65	2.26±1.96	0.00±0.00	0.81±1.57	0.16±0.74
LVD	-	-	-	3.03±2.02	0.00±0.00	1.41±2.51	1.07±2.61
**HBeAg**							
Positive	4.51±0.65	4.69±1.35	3.66±1.80	2.87±1.92	0.97±1.74	2.863±2.48	0.93±2.11
Negative	3.87±0.94	3.62±1.40	1.37±2.01	1.56±2.03	0.44±1.22	0.09±0.51	0.17±0.79
**VR at 48w**							
True	4.09±0.78	3.66±1.44	1.47±1.88	0.99±1.83	0.31±0.92	0.62±1.60	0.19±0.88
False	4.45±0.87	4.91±1.22	3.84±1.99	2.92±1.88	1.71±2.22	2.40±2.48	0.96±2,11
**HBsAg clearance**							
True	3.34±0.78	3.56±1.75	0.00±0.00	0.34±0.76	0.00±0.00	0.00±0.00	0.00±0.00
False	4.26±0.82	4.12±1.45	2.65±2.21	2.16±2.11	0.67±1.49	1.50±2.26	0.50±1.55

Supplementary Table 3 Clinical characteristics of HBV pgRNA positive and negative patients at week 48

	**Total (n=71)**	**HBV pgRNA negative (n=64)**	**HBV pgRNA positive (n=7)**	**P value**
**ALT(IU/L)**	27±16.0	28±16.8	21±11.62	>0.05
**HBV DNA (log10IU/mL)**	2.74±2.10	2.59±2.03	4.06±0.98	>0.05
**HBsAg (log10IU/mL)**	3.17±1.14	3.16±1.14	3.92±0.37	>0.05
**ALT normalization**	61/63	57/58	4/5	>0.05
**Undetectable HBV DNA**	19/71	19/64	0/7	>0.05
**HBeAg clearance**	8/51	8/45	0/6	>0.05
